# Metabolomics Analysis Reveals the Differences Between *Bupleurum chinense* DC. and *Bupleurum scorzonerifolium* Willd.

**DOI:** 10.3389/fpls.2022.933849

**Published:** 2022-07-13

**Authors:** Xuejie Qu, Shanqun Hu, Tong Li, Jiaqi Zhang, Baoshun Wang, Changli Liu

**Affiliations:** School of Traditional Chinese Medicine, Capital Medical University, Beijing, China

**Keywords:** *Bupleurum chinense* DC., *Bupleurum scorzonerifolium* Willd., differential metabolites, statistical analysis, saikosaponins

## Abstract

*Bupleurum chinense* DC. and *Bupleurum scorzonerifolium* Willd. are two varieties of Bupleuri Radix in Chinese Pharmacopoeia 2020. The clinical efficacy of the two bupleurum species is different. The difference in clinical efficacy is closely related to the composition of plant metabolites. In order to analyze the difference in metabolites, we used liquid chromatography coupled with mass spectrometry (LC-MS) for untargeted metabolome and gas chromatography coupled with mass spectrometry (GC-MS) for widely targeted metabolome to detect the roots (R), stems (S), leaves (L), and flowers (F) of two varieties, and detected 1,818 metabolites in 25 classes. We performed a statistical analysis of metabolites. Differential metabolites were screened by fold-change and variable importance in the projection values of the OPLS-DA model, and significant differences were found among different groups. The content of active components (triterpenoid saponins) was found to be high in the BcR group than in the BsR group. Other pharmacological metabolites were significantly different. By Kyoto Encyclopedia of Genes and Genomes annotation and enrichment analysis, we found that differential metabolites of the aboveground parts mainly concentrated in monoterpenoid biosynthesis, while the differential metabolites of the root mainly concentrated in sesquiterpenoid and triterpenoid biosynthesis. Differences in metabolic networks may indirectly affect the metabolic profile of Bc and Bs, leading to differences in clinical efficacy. Our study provides a scientific basis for subsequent biosynthesis pathway and related bioactivity research, and provides a reference for developing non-medicinal parts and guiding the clinical application of Bupleuri Radix.

## Introduction

Plants, as direct or indirect sources of nutrition, energy, and medicine for human beings, can synthesize a large number of metabolites with different biological functions under the changeable environmental stimulation. In the long evolutionary process, plants constantly mutate in order to adapt to changeable environments (Brunetti et al., 2013). They occupy wide habitats and usually vary in phenotypes, such as in morphology, and metabolism, thereby developing into different ecotypes. Plants contain primary and secondary metabolites. The latter are involved in the environmental response of resistance to disease and stress, and some substances have important medicinal values for humans (Yang et al., 2018). On a global scale, we recognize the importance of medicinal plants for humanity. They can supplement the nutrition needed by the human body, improve the immunity of the human body, improve the state of disease, and even fight epidemics, such as SARS-CoV-2 (Wang and Yang, 2021) and malaria (Talman et al., 2019). According to WHO reports, around 80% of the global population still relies on botanical drugs (Sen and Samanta, 2015). Natural substances have long served as sources of therapeutic drugs.

*Bupleurum* species are found mainly in the subtropical regions of the Northern hemisphere (Huang K. et al., 2021). The dry roots are used as herbal medicine in many countries, and some species have been officially listed in the Chinese, Japanese, Korean, British, and European Pharmacopoeias (Ashour and Wink, 2011; Ting-Ting et al., 2020). These species are used either alone or in combination with other ingredients for the treatment of common cold, inflammatory disorders, hepatitis, and fever (Moses et al., 2014). Up to now, phytochemical studies have shown that *Bupleurum* contains abundant natural compounds, such as flavonoids, lignins, phenolics, phenyl propanol derivatives, triterpenoid saponins, and volatile oils (Sun et al., 2019; Jiang H. et al., 2020). In the 2020 edition of the Chinese Pharmacopoeia, *Bupleurum chinense* DC. and *Bupleurum scorzonerifolium* Willd. were included as the two authentic sources of Chinese traditional medicine Bupleuri Radix (BR, chaihu). BR has been recorded in the Shennong Traditional Herbal Scriptures under its original name of Purput and was listed among the top herbs. It was renamed chaihu in the Book of Materia Medica and has been used for more than 2,000 years (Deborde and Jacob, 2014; Pavlidis et al., 2021). This herb is known to disperse and reduce fever, relieve liver depression, and lift yang-qi. The main active components of bupleurum are triterpenoid saponins, and specifically, saikosaponins (SSs) (Li et al., 2018) constitute a research hotspot in the field of plant medicine (He et al., 2021; Sui et al., 2021). In China, from ancient to the present, the efficacy of the two bupleurum species is different. Shiyuan Jin, a master of traditional Chinese medicine, said the use of two types of bupleurum in Beijing required doctors to write prescriptions separately according to their needs. *B. chinense* DC., commonly known as Bupleuri Chinense Radix, is often used to treat typhoid fever, while *B. scorzonerifolium* Willd., commonly known as Bupleuri Scorzonerifolii Radix, is often used to clear liver heat, so they exhibit clinical uses. The difference in clinical efficacy is closely related to the composition of plant metabolites.

The growing demand for medicinal plants has led to a short supply of resources. In order to improve the utilization of plants and alleviate resource shortages, researchers developed other tissue parts of medicinal plants (Liu et al., 2020). Different tissue parts have different compositions, and the curative effect is different (Maqbool et al., 2019). The official medicinal part of the bupleurum is the root. “Qianjin Yifang” recorded the use of bupleurum seedlings for treating sudden deafness, so the stems and leaves of bupleurum were also used at that time to treat diseases, and their effects were different from that of the root. Currently, the standard of local medicine in China includes the whole herb of *B. scorzonerifolium* Willd. Studies have found that the stems, leaves, and flowers of *B. chinense* DC. contain a large amount of flavonoids (Zhang et al., 2010; Cong et al., 2021). The development of the aboveground part of bupleurum is beneficial to saving resources and protecting ecology. This study compared the metabolomics composition of different tissue parts of *B. chinense* DC. and *B. scorzonerifolium* Willd., so as to provide a reference for utilizing other medicinal parts and guiding the clinical use of traditional Chinese medicine.

## Materials and Methods

### Plant Materials

In this study, 12 wild *B. chinense* DC. (Bc) and 12 wild *B. scorzonerifolium* Willd. (Bs) (at least 1 year old) were selected from the suburban mountainous areas of Beijing, China, in July 2021. Then the plants were harvested, rinsed with running water, and again rinsed with sterile water. The roots (R), stems (S), leaves (L), and flowers (F) were separated with a blade on a clean table, sampled, and stored at −80°C until further analysis. A total of eight groups (BcR, BcS, BcL, BcF, BsR, BsS, BsL, and BsF) were identified, with each sample comprising three biological replicates, and each replicate containing three individual plants. Fewer samples were taken because there were fewer plants of wild bupleurum in the mountain.

### Sample Preparation and Extraction

The samples were dried in a vacuum freeze-drying machine (ScientZ-100F). The tissue was ground to powder by using a grinder (MM 400; Retsch, Haan, Germany) for 1.5 min at 30 Hz. Then, 100 mg of powder was weighed, dissolved in 1.2 mL of 70% aqueous methanol, vortexed six times for 30 s each, and kept overnight at 4°C. After centrifugation at 12,000 rpm for 10 min, the supernatant was separated and filtered through microporous membranes (0.22 μm pore size, ANPEL, Shanghai, China) and stored in vials, and was followed by UPLC-MS analysis (Chen et al., 2013; Peng et al., 2017; Zhu et al., 2018).

Another portion of the sample was ground with liquid nitrogen and mixed uniformly by the vortex method. About 1 g of each sample was weighed in a headspace bottle. Then 2 mL of saturated NaCl solution and 10 μL (50 μg/mL) of the internal standard solution were added, respectively. Extraction was carried out by automatic headspace solid-phase microextraction (HS-SPME) for gas chromatography coupled with mass spectrometry (GC-MS) analysis.

### Ultra-Performance Liquid Chromatography and Gas Chromatography Conditions

The extracts were analyzed by liquid chromatography coupled with mass spectrometry (LC-MS) system (HPLC, UFLC SHIMADZU Nexera X2; MS, 4500 QTRAP; Applied Biosystems, Foster City, CA, United States) equipped with a C18 column (Agilent SB-C18, 2.1 mm × 100 mm, 1.8 μm). The mobile phase included ultra-pure water (A) and acetonitrile (B) with 0.1% acetic acid. The elution gradient procedure was performed as follows: 95:5 V/V for 0 min, 5:95 V/V at 9.0 min, 5:95 V/V at 10.0 min, 95:5 V/V at 11.1 min, and 95:5 V/V at 14.0 min. The flow rate was 0.35 mL/min. The column temperature was set at 40°C, and the injection volume was 4 μL. The effluent was connected to an ESI-triple quadrupole linear ion trap mass spectrometer (Q TRAP)-MS (Zhang et al., 2020).

Desorption of the volatile organic chemicals (VOCs) from the fiber coating was carried out in the injection port of the GC apparatus (Model 8890; Agilent) at 250°C for 5 min in the splitless mode. GC apparatus equipped with a DB-5MS capillary column (30 m × 0.25 mm × 0.25 μm, Agilent J&W Scientific, Folsom, CA, United States) was used. Helium was used as the carrier gas at a linear velocity of 1.2 mL/min. The injector temperature was kept at 250°C and the detector at 280°C. The oven temperature was programmed from 40°C (3.5 min), increasing at 10°C/min, to 100°C, at 7°C/min to 180°C, at 25°C/min to 280°C, and held for 5 min. The effluent was connected to an electron impact (EI)-MS.

### ESI-Q TRAP-MS/MS and Electron Impact-Mass Spectrometry Conditions

In order to detect metabolites, AB4500 Q TRAP LC/MS/MS system equipped with linear ion trap (LIT) and triple quadrupole (QQQ) scans was operated. This system was equipped with an ESI turbo ion spray interface operating in positive and negative ion monitoring mode and controlled by Analyst v1.6.3 software (AB Sciex, Foster City, CA, United States). The ESI source was an ion source (turbo spray, 550°C, 5,500 V). Gas I, gas II, and curtain gas (CUR) were set at 50, 60, and 25 psi, respectively. The collision-activated dissociation was high. In the QQQ and LIT modes, 10 and 100 μmol/L of polypropylene glycol solutions, respectively, were used for the instrument tuning and mass calibration. QQQ scans were obtained in the multiple reaction monitoring (MRM) experiments with the collision gas (nitrogen) set to 5 psi. By further optimizing de-clustering potential (DP) and collision energy (CE), the DP and CE of each MRM ion pair were completed. According to the eluted metabolites in each period, a specific set of MRM transitions was monitored.

The GC-MS spectrum was recorded in EI ionization mode at 70 eV. The quadrupole mass detector, ion source, and transfer line temperatures were set at 150, 230, and 280°C, respectively. The MS in selected ion monitoring (SIM) mode was used for the identification and quantification of analytes.

### Qualitative and Quantitative Metabolite Analyses

For LC-MS, we compared the accurate precursor ions (Q1), product ion (Q3) values, retention times (RTs), and fragmentation patterns with the standards to analyze the primary and secondary MS data, when standards were available (Sigma-Aldrich, St. Louis, MO, United States). When standards were unavailable, we used the MWDB (MetWare Biological Science and Technology Co., Ltd., Wuhan, China), which is a self-compiled database, to analyze the data. Qualitative analysis was carried out according to secondary spectrum information, and subsequently isotope signals, repeated signals containing K+, Na+, and NH4+, and repeated signals of fragments of other substances with a larger molecular weight were removed during analysis.

The quantitative analysis was carried out in the MRM mode. The characteristic ions of each metabolite were screened through the QQQ MS to obtain the signal strengths. Integration and correction of chromatographic peaks were conducted using MultiQuant v3.0.2 (AB Sciex, Concord, ON, Canada). The corresponding relative metabolite contents were expressed as chromatographic peak area integrals.

For GC-MS, we used a self-compiled database MWGCSIM1.0 (MetWare Biological Science and Technology Co., Ltd., Wuhan, China). Each compound matched a quantitative ion and 2–3 qualitative ions. The RT of the detected chromatographic peak was consistent with the standard reference, and all the selected ions appeared in the quality spectrum of the sample after deducting the background, then the substance was determined (Yuan et al., 2022). Quantitative ions were selected to integrate and correct chromatographic peaks to enhance the accuracy of quantification. Integration and correction of chromatographic peaks were conducted using MassHunter B.08.00.

In order to keep the experimental data stable, we carried out a quality control analysis of the sample before testing the sample.

### Multivariate Statistical Analysis

Metabolite data were log_2_-transformed for statistical analysis to improve normality and were normalized. Metabolites from 24 samples were used for hierarchical clustering analysis (HCA) within R (ComplexHeatmap 2.8.0), principal component analysis (PCA) within R (base package 3.5.1), and orthogonal partial least squares discriminant analysis (OPLS-DA) within R (MetaboAnalystR 1.0.1). Venn diagrams were used to illustrate the number of differential metabolites. Identified metabolites were annotated using the Kyoto Encyclopedia of Genes and Genomes (KEGG) compound database,^1^ and the annotated metabolites were then mapped to the KEGG pathway database.^2^

## Results

### Morphological Differences

**FIGURE 1:**
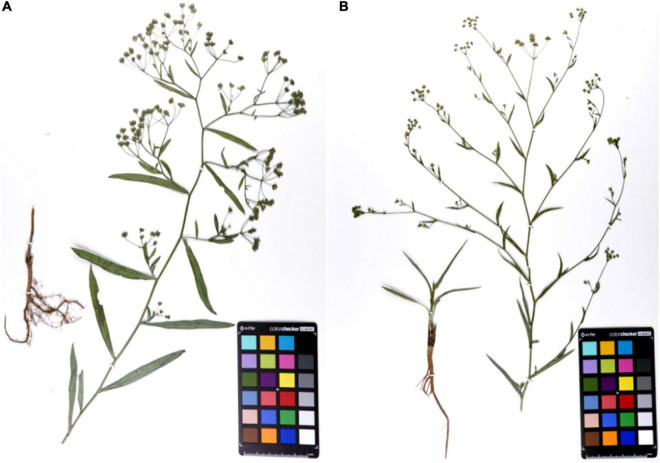
The specimens of mature *Bupleurum chinense* DC. **(A)** and *Bupleurum scorzonerifolium* Willd. **(B)**.

*Bupleurum chinense* DC. and *B. scorzonerifolium* Willd. (Figure 1) were grown in the same season and under similar conditions. The morphology of the root in particular was obviously different. The branches of Bc outnumbered those found in Bs. The skin color of Bc was chocolate-brown, but that of Bs was reddish-brown. In addition, the leaves of Bs were narrowly elliptic or oblanceolate with 7–9 parallel veins, while the leaves of Bs were linear-lanceolate with 5–7 parallel veins. The compound umbels of Bs have significantly more small umbrella stems than the umbels of Bc.

### Metabolic Profiling

**FIGURE 2:**
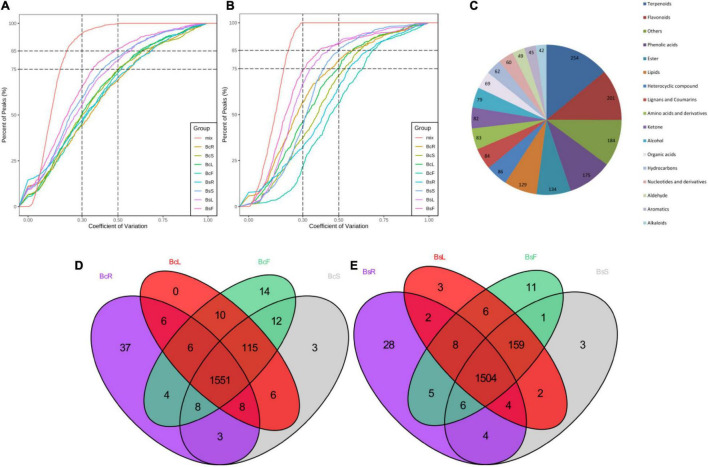
Quality control (QC) sample with the coefficient of variation (CV) of LC-MS **(A)** and GC-MS **(B)** less than 0.3 accounted for more than 75% of the samples, indicating that the experimental data were very stable. Overview of annotated metabolites **(C)**. Venn diagram of the detected metabolites in four tissues of Bc **(D)** and Bs **(E)**.

In order to understand more clearly the metabolites that occur in different tissue parts of Bc and Bs, primary and secondary metabolites were identified by UPLC-MS/MS and GC-MS (Figures 2A,B). A total of 1,818 metabolites were detected, including 254 terpenoids, 201 flavonoids, 175 phenolic acids, and other compounds (Figure 2C). A total of 1,733, 1,776, 1,782, and 1,784 metabolites were detected in the four contrast groups of roots, stems, leaves, and flowers, respectively (Figures 2D,E). In general, this result indicated that there were different metabolic profiles in different tissues. Terpenoids were the main medicinal compounds in *Bupleurum*. Around 254 terpenoids were detected, including 30 triterpenoid saponins, 11 monoterpenoids, 5 diterpenoids, 19 triterpenoids, and 3 sesquiterpenoids.

### Multivariate Analysis Revealed Differences Among the Metabolite Profiles

**FIGURE 3:**
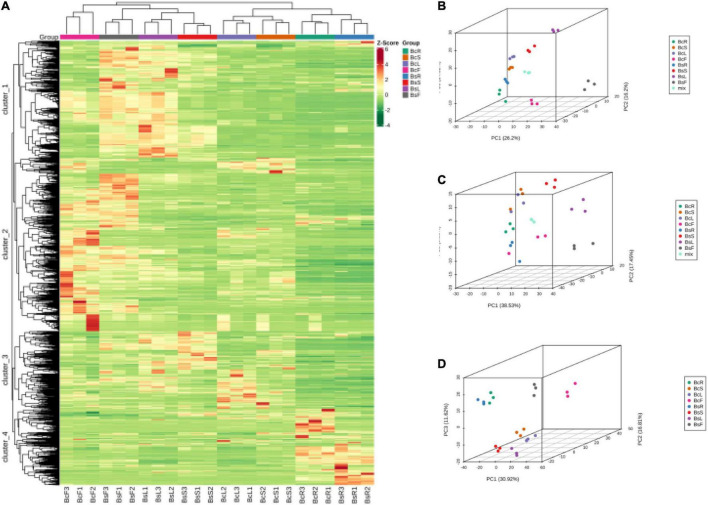
Heat map of HCA and PCA of the relative differences in the metabolite profiles between different varieties and parts. The metabolites are divided into four clusters **(A)**, 3D PCA plot of LC-MS **(B)**, GC-MS **(C)**, and incorporated PCA plot **(D)**.

Multivariate statistical analysis was performed to assess the differences between the metabolite profiles of eight groups. The results of HCA showed that there were significant differences in different groups, which were divided into four clusters (Figure 3A). The metabolites in cluster 1 were highest in BsF and BsL, cluster 2 in BcF and BsF, cluster 3 in BsS and BcL, and cluster 4 in BcR and BsR. It also clustered between biological replicates, indicating good homogeneity between biological replicates and reliability of data.

Principal component analysis was used to uncover the internal structure of multiple variables through several principal components. In the PCA diagram (Figures 3B–D), the QC sample is the mixture of bupleurum sample extracts, which were projected to the same area and some even overlapped, so the analysis was stable and repeatable. This result showed that the tissue sites were separated on the first principal component, and the varieties showed obvious separation on the second principal component. There was an obvious grouping tendency among different tissues of different varieties.

In general, HCA and PCA showed that there were significant differences in metabolites in different groups, with BcR and BsF showing the biggest differences.

### Differential Metabolite Analysis

**FIGURE 4:**
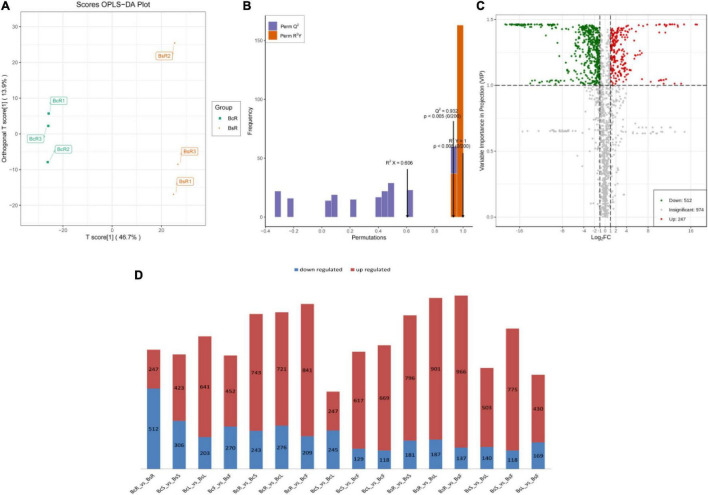
The groups of BcR and BsR are shown, including the score OPLS-DA plot **(A)**, OPLS-DA model simulation verification diagram **(B)**, and volcano plots of differential metabolites **(C)**. The statistical analysis of the number of differential metabolites for all different groups is presented **(D)**, with the red color indicating upregulated metabolites and the blue color indicating downregulated metabolites.

Pairwise comparisons were conducted among the eight groups (Figure 4D). In the OPLS-DA models (Figures 4A,B), significant segregation occurred in all comparison groups.

**FIGURE 5:**
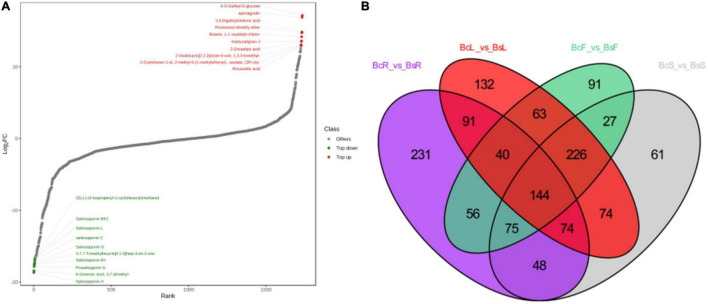
The top 10 downregulated and upregulated metabolites in the BcR and BsR groups **(A)**. Venn diagram between different tissues showed the overlapping and accession-specific differential metabolites **(B)**.

We further performed differential metabolite screening based on the fold-change (FC ≥2 or ≤0.5) and variables identified as important in the projection scores (VIP >1). The screening results were presented as volcano plots (Figure 4C) and Venn diagrams (Figure 5B).

Among all comparison groups, BcR and BsR were downregulated the most, which was consistent with the results of the cluster analysis. We paid specific attention to the differential metabolites in the root group. There were 759 differential metabolites, of which 247 metabolites were upregulated and 512 metabolites were downregulated. There were 150 terpenoids in the differential metabolites, accounting for about 20% (Table 1).

**TABLE 1:** Classification and quantity of differential metabolites between BcR and BsR groups.

Type	Number	Percentage
Terpenoids	150	19.8
Phenolic acids	73	9.6
Flavonoids	72	9.5
Lipids	44	5.8
Lignans and coumarins	43	5.7
Hydrocarbons	27	3.6
Alkaloids	15	2.0

In the BcR and BsR groups, 7 of the top 10 downregulated saponins were SSs (Figure 5A), such as saikosaponin A, prosaikogenin G, saikosaponin B1, saikosaponin G, saikosaponin C, saikosaponin L, and saikosaponin BK1. Basically, all saponin metabolites in Bs were downregulated, indicating that the content of SSs in the root of Bc was relatively high. The top 10 downregulated metabolites included lignans, phenolic acids, and volatile organic chemicals. More amount of SSs (SSA, SSC, SSD, SSB4, and SSF), which have been shown to have antidepressant and anti-inflammatory effects, were detected in the BcR group than in the BsR group. It is worth noting that BsR has far more content of saponarin, prunetin, 3-*O*-acetylpinobanksin, methylhesperidin 6-*O*-galloyl-D-glucose, and epimagnolin than BcR. With regard to isorhamnetin, which has the cardiovascular and cerebrovascular protective, anti-inflammatory, anti-oxidation, organ protection, prevention of obesity, and other effects (Gong et al., 2020), we found that 16 isorhamnosides were more accumulated in BsR than in BcR.

The comparison between the BcS and BsS groups revealed 729 differential metabolites in total, among which 423 metabolites were upregulated and 306 metabolites were downregulated, and flavonoids were the most differential metabolites. The comparison between the BcL and BsL groups revealed 844 differential metabolites in total, among which 641 metabolites were upregulated and 203 metabolites were downregulated, and flavonoids and terpenoids were the main differential metabolites. The comparison between the BcF and BsF groups showed 722 differential metabolites in total, among which 452 metabolites were upregulated and 270 metabolites were downregulated. More amount of hispidulin-8-C-(2′′-*O*-xylosyl)glucoside was found in BcF than in BsF. More content of kaempferol-3-*O*-glucuronide, which had been used to treat non-alcoholic steatohepatitis (Deng et al., 2021), was found in BsF than in BcF.

We also compared differential metabolites between different tissue parts. In contrast groups of BcR and BcF, 841 metabolites were upregulated, including 149 flavonoids. In Bc, there were more triterpenoid saponins in roots, but more amounts of flavonoids and volatile components in flowers. In the contrast group of BsR and BsF, 966 metabolites were upregulated, including 109 flavonoids and 78 phenolic acids, and the number of upregulated metabolites was the largest in all contrast groups. So, we can develop the flowers of Bc and Bs to use active ingredients, such as flavonoids. In addition, there were more flavonoids and phenolic acids in the aboveground parts than in the root of Bs.

We identified 144 common differential metabolites from Venn diagrams. The common differential metabolites indicated that Bc and Bs showed differences in roots, stems, leaves, and flowers, indicating interspecific differential metabolites. We focused on triterpenoid saponins among all downregulated components in four contrast groups, such as oleanolic acid-3-*O*-xylosyl(1 → 3)glucuronide, oleanolic acid-3-*O*-glucosyl(1 → 2)glucoside, bupleurumwenchuanense, saikosaponin K, rotundifolioside A, rotundifolioside H, and medicagenic acid-3-*O*-glucuronide-28-*O*-rhamnosyl(1,2)-arabinoside. More triterpenoid saponins were synthesized in Bc than in Bs, which was closely related to the expression of genes related to the triterpenoid saponins synthesis pathway. The other downregulated metabolites mostly comprised flavones, flavonoid carbonoside, and flavonols, including eight kinds of kaempferitrins and apiins. All upregulated metabolites included 25 flavonoids, 11 phenolic acids, and some volatile components.

It is noteworthy that shikimic acid, as the prerequisite of phenylpropane pathway, was lower in all parts of Bs than that of Bc, which may be related to the metabolism of phenylpropane pathway in the two varieties. Isoscopoletin (6-hydroxy-7-methoxycoumarin), which showed anti-asthmatic effects, was higher in Bc than in Bs.

### Kyoto Encyclopedia of Genes and Genomes Annotation and Enrichment Analysis

**FIGURE 6:**
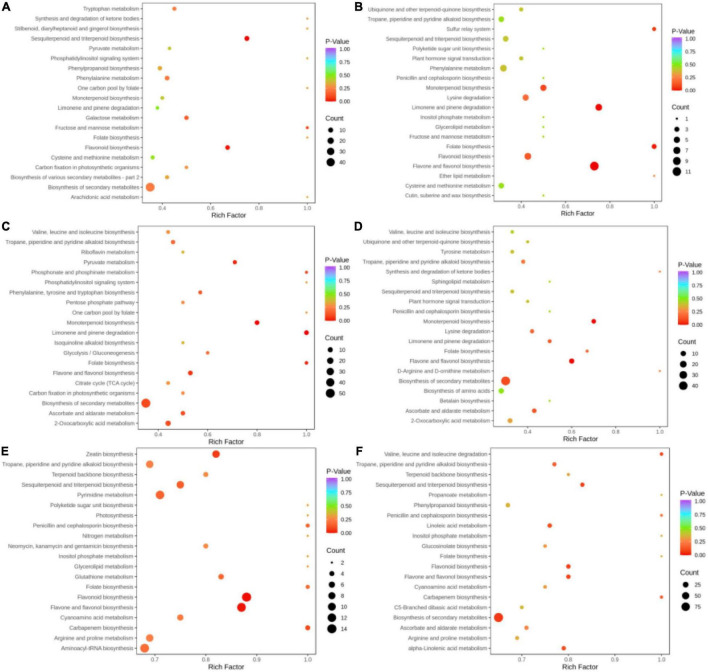
Differential metabolite pathway bubble map. **(A)** BcR and BsR. **(B)** BcS and BsS. **(C)** BcL and BsL. **(D)** BcF and BsF. **(E)** BcR and BcF. **(F)** BsR and BsF.

Pathway annotation and enrichment analysis by the KEGG pathway database were performed for key differential metabolites, and the top 20 significant pathways were related to 6 groups of metabolites (Figures 6A–F). In the BcR and BsR groups, differential metabolites were mainly enriched in sesquiterpenoid and triterpenoid biosynthesis, flavonoid biosynthesis, and biosynthesis of secondary metabolites. In the BcS and BsS groups, differential metabolites were mainly enriched in flavone and flavonol biosynthesis, limonene and pinene biosynthesis, monoterpenoid biosynthesis, and folate biosynthesis. In the BcL and BsL groups, differential metabolites were mainly enriched in monoterpenoid biosynthesis, limonene and pinene biosynthesis, and biosynthesis of secondary metabolites. In the BcF and BsF groups, differential metabolites were mainly enriched in monoterpenoid biosynthesis, flavone and flavonol biosynthesis, and biosynthesis of secondary metabolites.

Some metabolic pathways overlapped in these comparison groups, such as biosynthesis of secondary metabolites and monoterpenoid biosynthesis. The differential metabolites of the aboveground parts of the two species mainly concentrated in monoterpenoid biosynthesis, while the differential metabolites of roots were mainly related to the sesquiterpenoid and triterpenoid biosynthesis. The differences in functions between the two species can be explained by the metabolic pathway, which is mainly due to the differences in the biosynthesis of terpenoids.

In the BcR and BcF groups, differential metabolites were mainly enriched in flavonoid biosynthesis, flavone and flavonol biosynthesis, pyrimidine metabolism, zeatin biosynthesis, and sesquiterpenoid and triterpenoid biosynthesis. Among these pathways, the flavonoid biosynthesis pathway in flowers deserves attention.

## Discussion

In the course of plant evolution, naturally occurring mutations are the primary source of phenotypic variation in plants. Natural selection influences standing genetic and phenotypic variations (Kalisz and Kramer, 2008), which explains the phenotypic differences in *Bupleurum*. The genetic variation in *Bupleurum* mainly affected the biosynthetic pathway of a compound in the plant, leading to the differences in the accumulation of that compound among different varieties (Liu et al., 2015). Therefore, the metabolome of mature *B. chinense* DC. and *B. scorzonerifolium* Willd. is significantly different.

We used LC-MS and GC-MS to analyze the roots, stems, leaves, and flowers of mature Bc and Bs, and detected a total of 1,818 metabolites belonging to 25 classes. We performed the statistical analysis of metabolites, and significant differences were found among different groups (BcR, BcS, BcL, BcF, BsR, BsS, BsL, and BsF). We also focused on some of the more diverse pharmacological components.

Triterpenoid saponins possess a 30C oxidosqualene precursor-based aglycone moiety (sapogenin) (Biswas and Dwivedi, 2019). Plant triterpenoid aglycones are further glycosated at the hydroxyl or carboxyl groups, which is catalyzed by a class of enzymes known as UDP-glycosyltransferases (UGTs) (Seki et al., 2015). Variation and differential expression of key enzyme genes in the biosynthetic pathway of triterpenoid saponins can affect the type and yield of saponins in plants (Fan et al., 2021). The spatial and temporal regulation of enzymes during development and in response to biotic and abiotic factors contribute to the time-variable formation of a diverse group of terpene metabolites (Tholl, 2006). Triterpenoid saponins have been shown to exhibit various medicinal properties, such as anti-inflammatory (Yang et al., 2021), anti-bacterial (Yu et al., 2019), anti-allergic (Han and Kim, 2020), anti-tumor (Han et al., 2019), immunomodulatory (Sarikahya et al., 2018), anti-carcinogenic (Li et al., 2021; Zhou et al., 2021), and hepatoprotective effects (Shao et al., 2021). In our study, BcR has more triterpenoid saponins as the main bioactive component than BsR. Combined with previous studies, we speculate that the huge differences in the content of triterpenoid saponins, particularly SSs, are closely related to clinical effects.

Flavonoids, which contribute to plant growth and development, are derived from the phenylpropanoid metabolic pathway and have a basic structure that comprises a C15 benzene ring structure of C6–C3–C6 (Liu W. et al., 2021). The pharmacological study indicates that flavonoids in BR exhibit antioxidant, bacteriostatic, and hepatoprotective properties (Jiang H. et al., 2020). In our study, we found significantly increased amounts of saponarin, prunetin, 3-*O*-acetylpinobanksin, methylhesperidin, and other flavonoids in BsR than in BcR. Saponarin, which is the sole flavonoid present in barley sprout leaves (Kim et al., 2022), possesses potent antioxidant (Kamiyama and Shibamoto, 2012) and hepatoprotective activities (Simeonova et al., 2013), and can be potentially used to prevent and relieve immune-related skin diseases (Min et al., 2021). Prunetin has anti-inflammatory effects (Abusaliya et al., 2022), prevents hepatocellular carcinogenesis by improving functions of the liver (Li G. et al., 2022), and suppresses high-fat diet (HFD)-induced adipogenesis in the liver tissues of mice by regulating gene expression in liver tissue (Ahn et al., 2013). 3-*O*-acetylpinobanksin also shows antioxidant activity (Jiang X. et al., 2020). Methylhesperidin may contribute to the suppression of UVB-induced skin damage *in vivo* (Kuwano et al., 2015).

There are also other types of compounds that have pharmacological activity. 6-*O*-galloyl-D-glucose in BsR outnumber it in BcR, which is phenolic acid and has antihypertensive activity (Hsu et al., 1994). Epimagnolin in BsR outnumber it in BcR, which is lignan and is used to treat nasal congestion, headache, and sinusitis in Asian countries (Baek et al., 2009; Yoo et al., 2019). The presence of more amounts of these metabolites in BsR than in BcR tended to strengthen the liver-soothing effects of Bupleuri Scorzonerifolii Radix. The differences in the types and contents of these pharmacologically active metabolites could affect the pharmacological action and clinical efficacy of Radix Bupleuri.

Studies have shown that bupleurum overground parts also have certain effective constituents (Liu Y. et al., 2021), and the development of non-medicinal parts is beneficial to saving resources (Chen and Yang, 2018) and protecting ecology (Zhang et al., 2022). The triterpenoid saponins were more in BcR than in BcF, while the flavonoids and VOCs were more in BcF. Some of these results are consistent with those of other studies (Li H. M. et al., 2022). From the perspective of plant biosynthesis, the contents of different tissues were consistent with the expression of key enzyme genes in the synthetic pathway, and the differential expression of key enzyme genes regulates the synthesis and accumulation of flavonoids and triterpenoid saponins in different tissues (Yang et al., 2019; Yu et al., 2020). In addition, we also found a lot of active ingredients in the stems and leaves, which show certain differences between Bc and Bs species. The aboveground part is rich in active ingredients and has great value for development and utilization.

By KEGG annotation and enrichment analysis, we found that differential metabolites of the aboveground parts of the two species mainly concentrated in the biosynthetic pathways of monoterpenoids, while differential metabolites of the root mainly concentrated in the sesquiterpenoid and triterpenoid biosynthesis. Differential metabolites of different bupleurum species involve different metabolic pathways, and these metabolic pathways depend on each other to maintain the physiological activities of organs. For example, terpene biosynthesis has been reported to be associated with glycolysis and the pentose phosphate pathway (Muhlemann et al., 2012). Mevalonic acid biosynthesis provides a precursor compound for terpene biosynthesis (Liao et al., 2016). The differences in metabolic networks may firsthand affect the metabolic profile of Bc and Bs, leading to differences in the clinical efficacy. Different tissues of plants show different functions, and their metabolic pathways are also different. The differences between tissues are related to the differential expression of genes and also involve the transport and accumulation of metabolites between different tissues.

The complex chemical components of medicinal plants affect the clinical efficacy of traditional Chinese medicine (Huang R. et al., 2021), particularly the active ingredients. The difference in metabolites between *B. chinense* DC. and *B. scorzonerifolium* Willd. resulted in the difference in the clinical efficacy of Bupleuri Radix. The spatial and temporal regulation of genes affects the synthesis and accumulation of metabolites in different tissues of Bc and Bs. The study by metabolomics analysis may provide a scientific basis for subsequent biosynthesis pathways and related bioactivity research. The correlation between the type or content of components in *Bupleurum* and the specificity and universality of efficacy still needs further pharmacological verification. The metabolomics analysis of different tissue parts of Bc and Bs might provide a reference for guiding the clinical use of traditional Chinese medicine and the developing of other medicinal parts.

## Data Availability Statement

The original contributions presented in this study are included in the article/Supplementary Material, further inquiries can be directed to the corresponding author.

## Author Contributions

XQ and CL conceived and designed the experiments, analyzed the data, and wrote the manuscript. XQ, SH, TL, and CL collected the plant material and performed the experiments. XQ, SH, TL, JZ, BW, and CL discussed the results and contributed to the drafting of the manuscript. All authors read and approved the final version of the manuscript.

## Conflict of Interest

The authors declare that the research was conducted in the absence of any commercial or financial relationships that could be construed as a potential conflict of interest.

## Publisher’s Note

All claims expressed in this article are solely those of the authors and do not necessarily represent those of their affiliated organizations, or those of the publisher, the editors and the reviewers. Any product that may be evaluated in this article, or claim that may be made by its manufacturer, is not guaranteed or endorsed by the publisher.

## Footnotes

1
http://www.kegg.jp/kegg/compound/

2
http://www.kegg.jp/kegg/pathway.html

## Abbreviations

Bc: *Bupleurum chinense* DC.
Bs: *Bupleurum scorzonerifolium* Willd.
BR: Bupleuri Radix
BcR: the root of *Bupleurum chinense* DC.
BsR: the root of *Bupleurum scorzonerifolium* Willd.
BcS: the stem of *Bupleurum chinense* DC.
BsS: the stem of *Bupleurum scorzonerifolium* Willd.
BcL: the leaf of *Bupleurum chinense* DC.
BsL: the leaf of *Bupleurum scorzonerifolium* Willd.
BcF: the flower of *Bupleurum chinense* DC.
BsF: the flower of *Bupleurum scorzonerifolium* Willd.
CV: coefficient of variation
CPS: counts per second
GC-MS: gas chromatography coupled with mass spectrometry
HCA: hierarchical clustering analysis
KEGG: Kyoto Encyclopedia of Genes and Genomes
LC-MS: liquid chromatography coupled with mass spectrometry
MS: mass spectrometry
OPLS-DA: orthogonal projections to latent structures-discriminate analysis
PCA: principal component analysis
QC: quality control
RT: retention time
SSs: saikosaponins
UHPLC: ultra-high performance liquid chromatography
VIP: variable importance in the projection.
