# Supplementary figures and images for: Metabolomics Analysis Reveals the Differences Between Bupleurum chinense DC. and Bupleurum scorzonerifolium Willd
Source: Front Plant Sci. 2022 Jul 13;13:933849. doi: 10.3389/fpls.2022.933849 (PMC9328751; doi:10.3389/fpls.2022.933849)

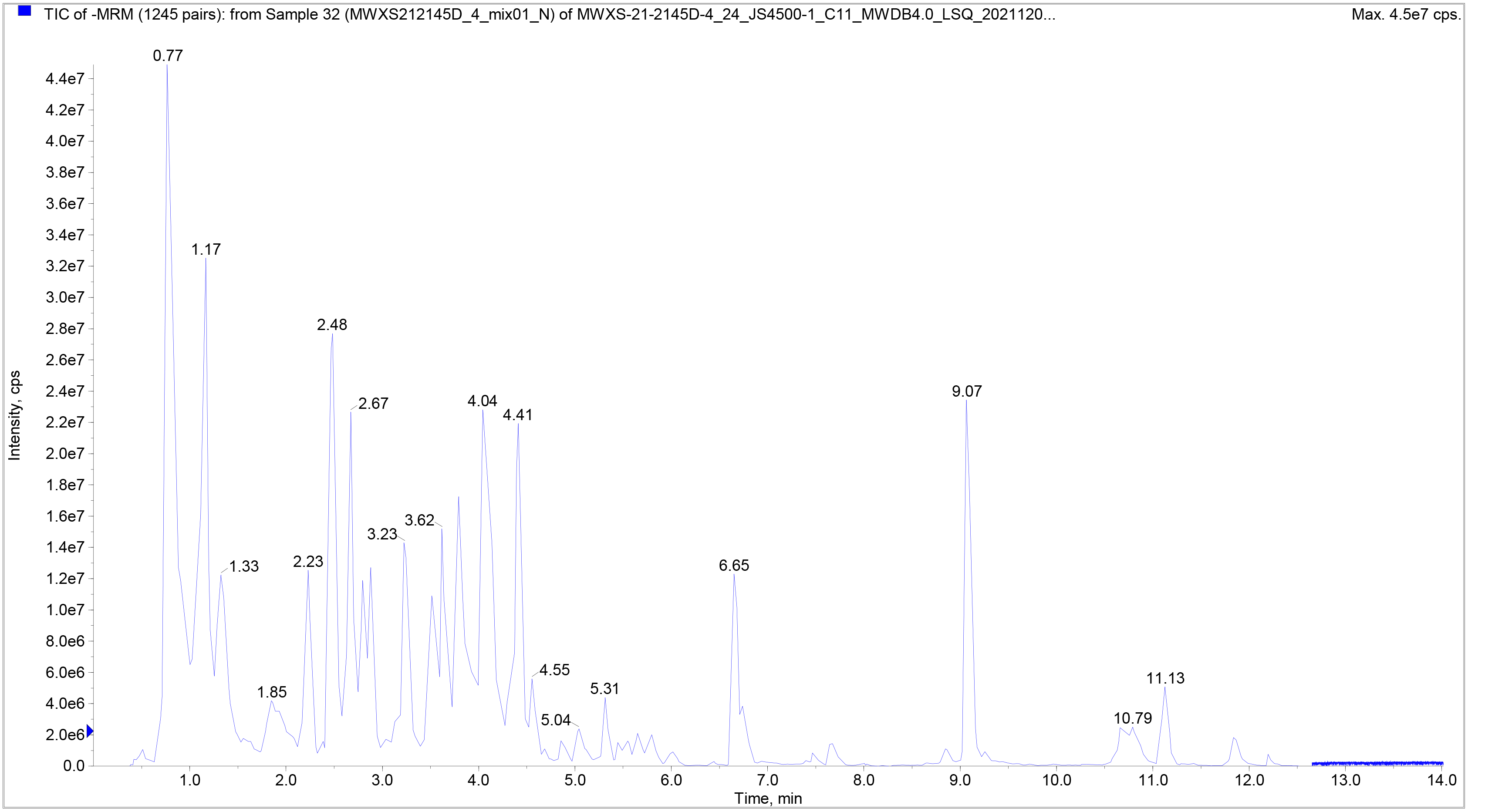

Supplement: Supplementary file 10 [file Image_1.TIF]

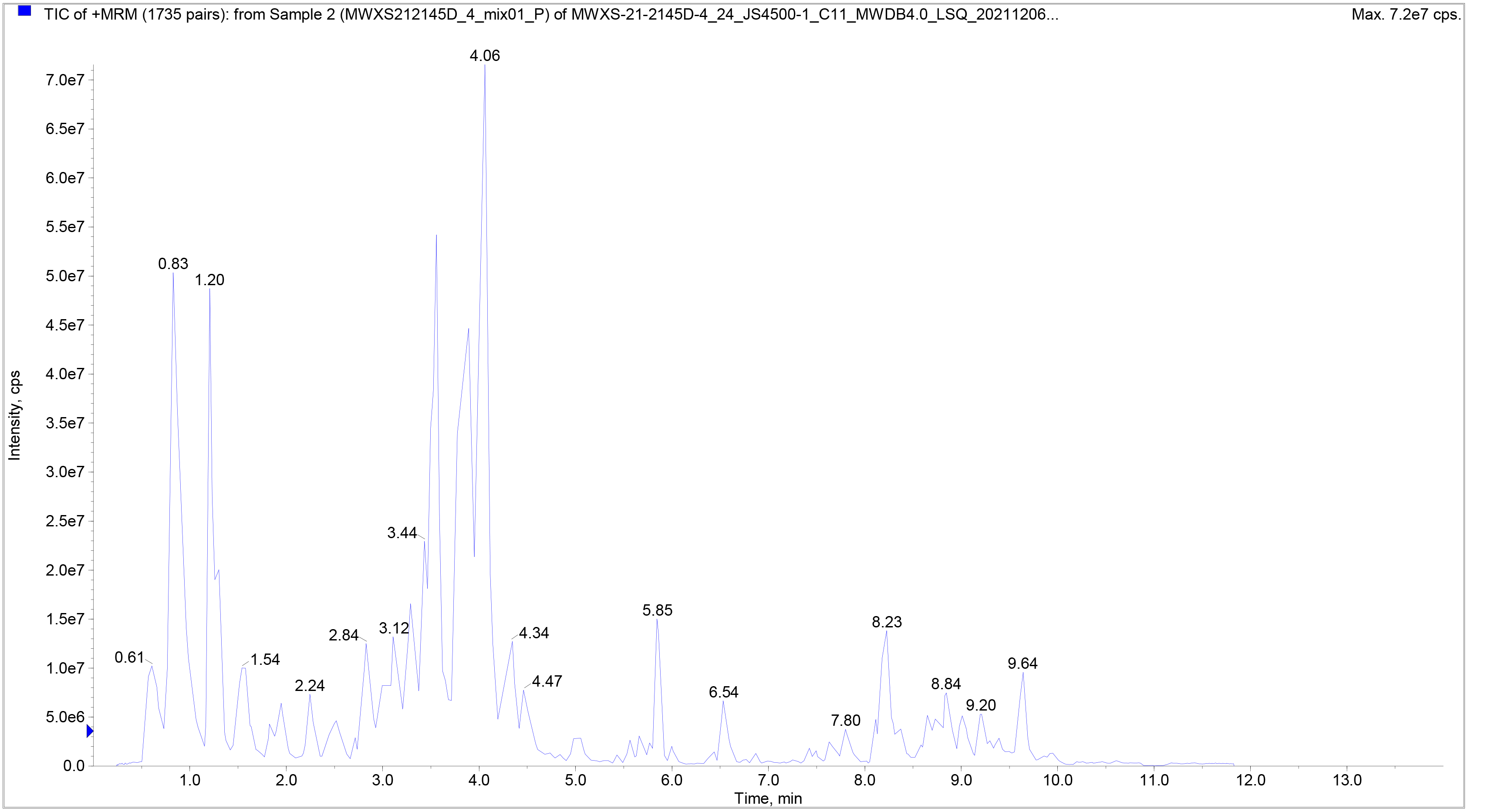

Supplement: Supplementary file 11 [file Image_2.TIF]

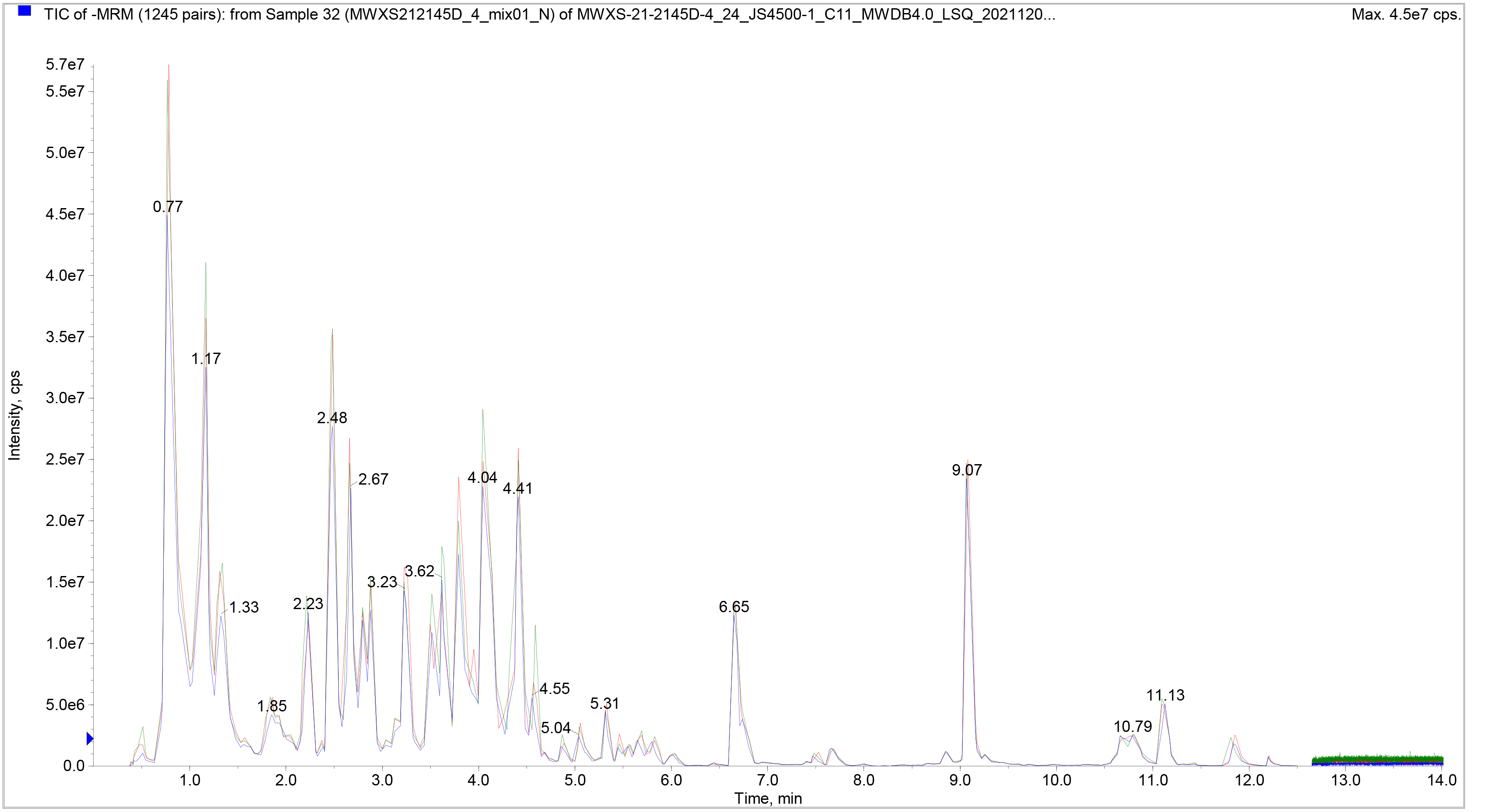

Supplement: Supplementary file 12 [file Image_3.TIF]

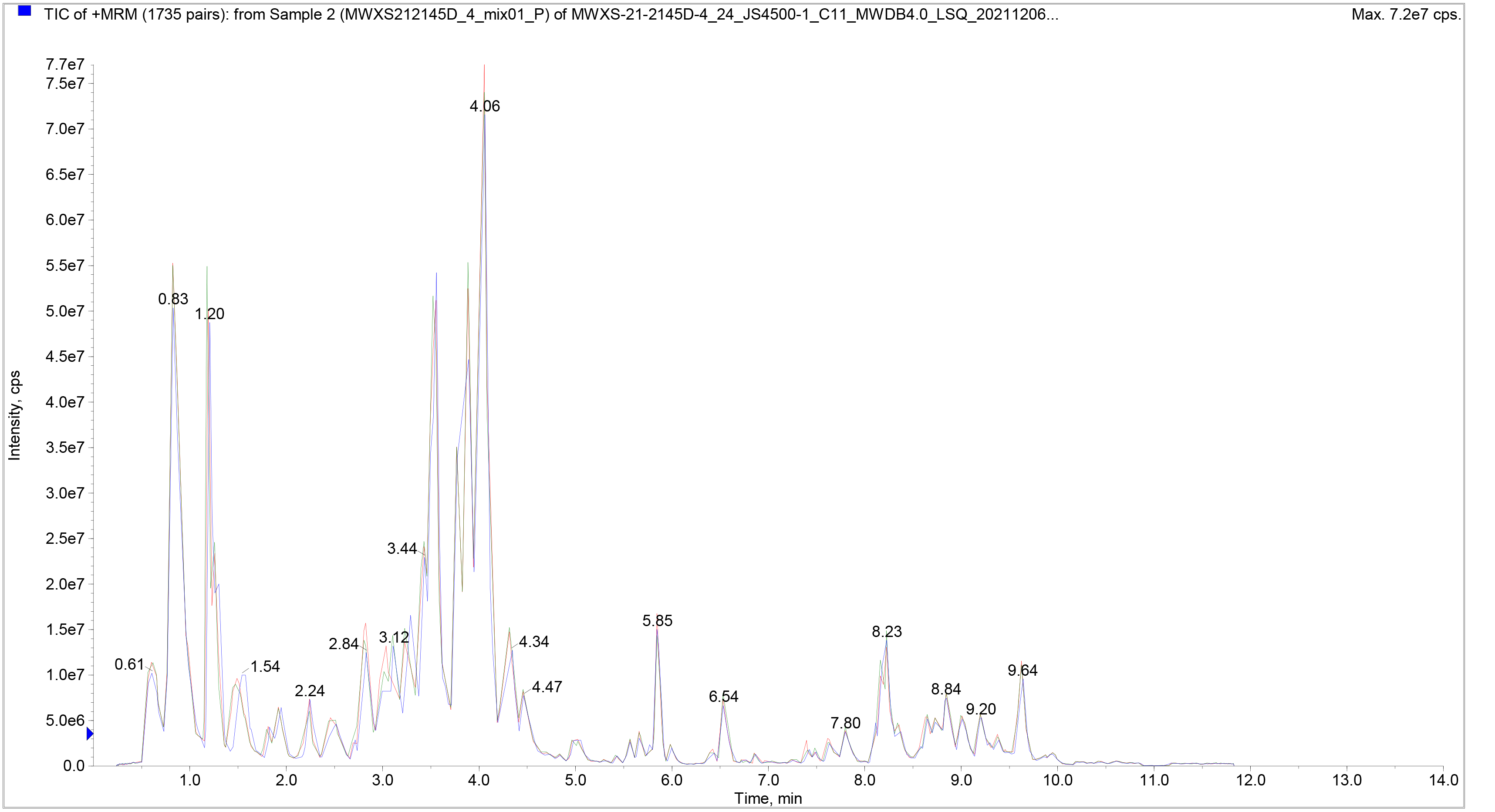

Supplement: Supplementary file 13 [file Image_4.TIF]

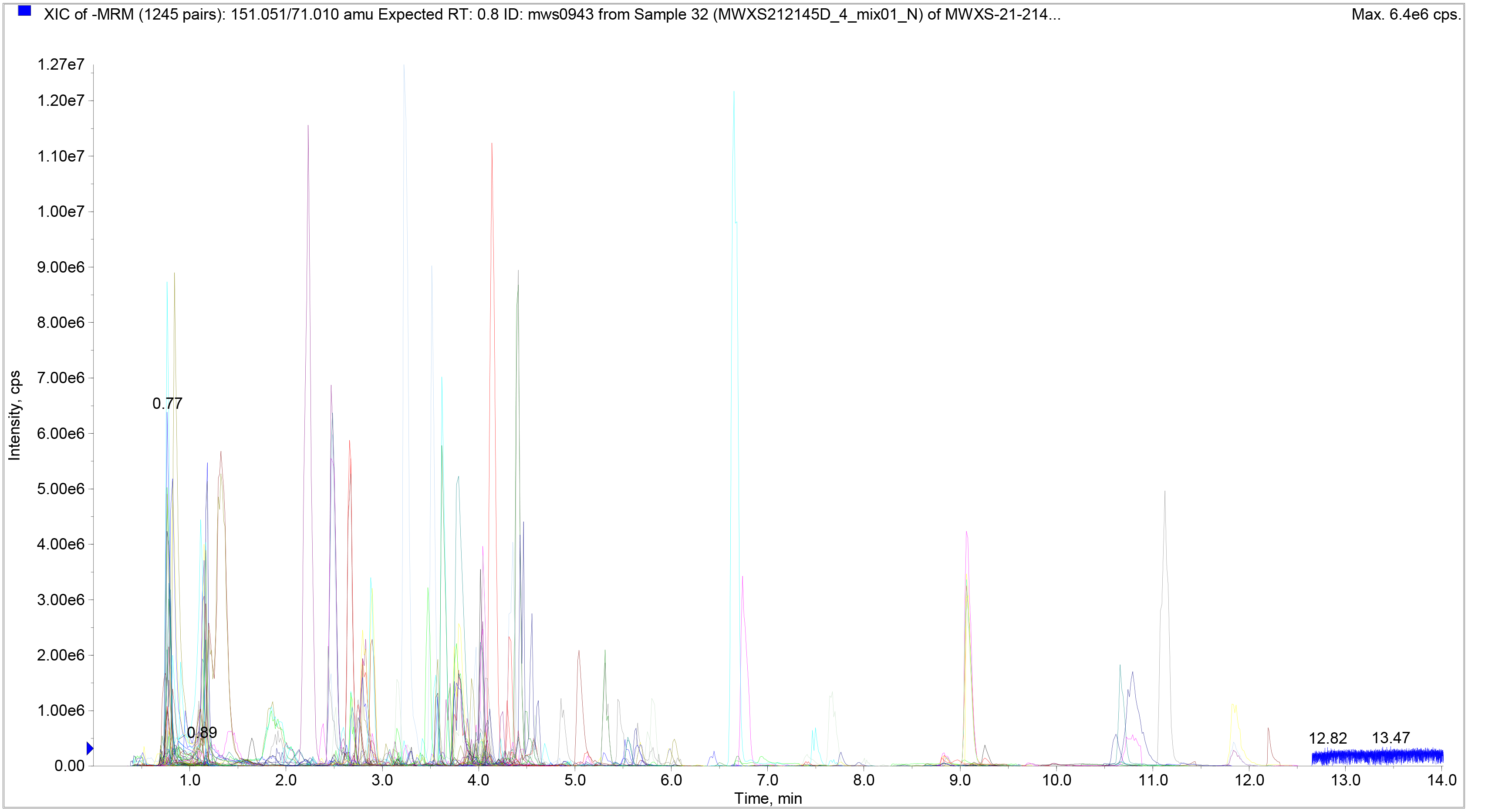

Supplement: Supplementary file 14 [file Image_5.TIF]

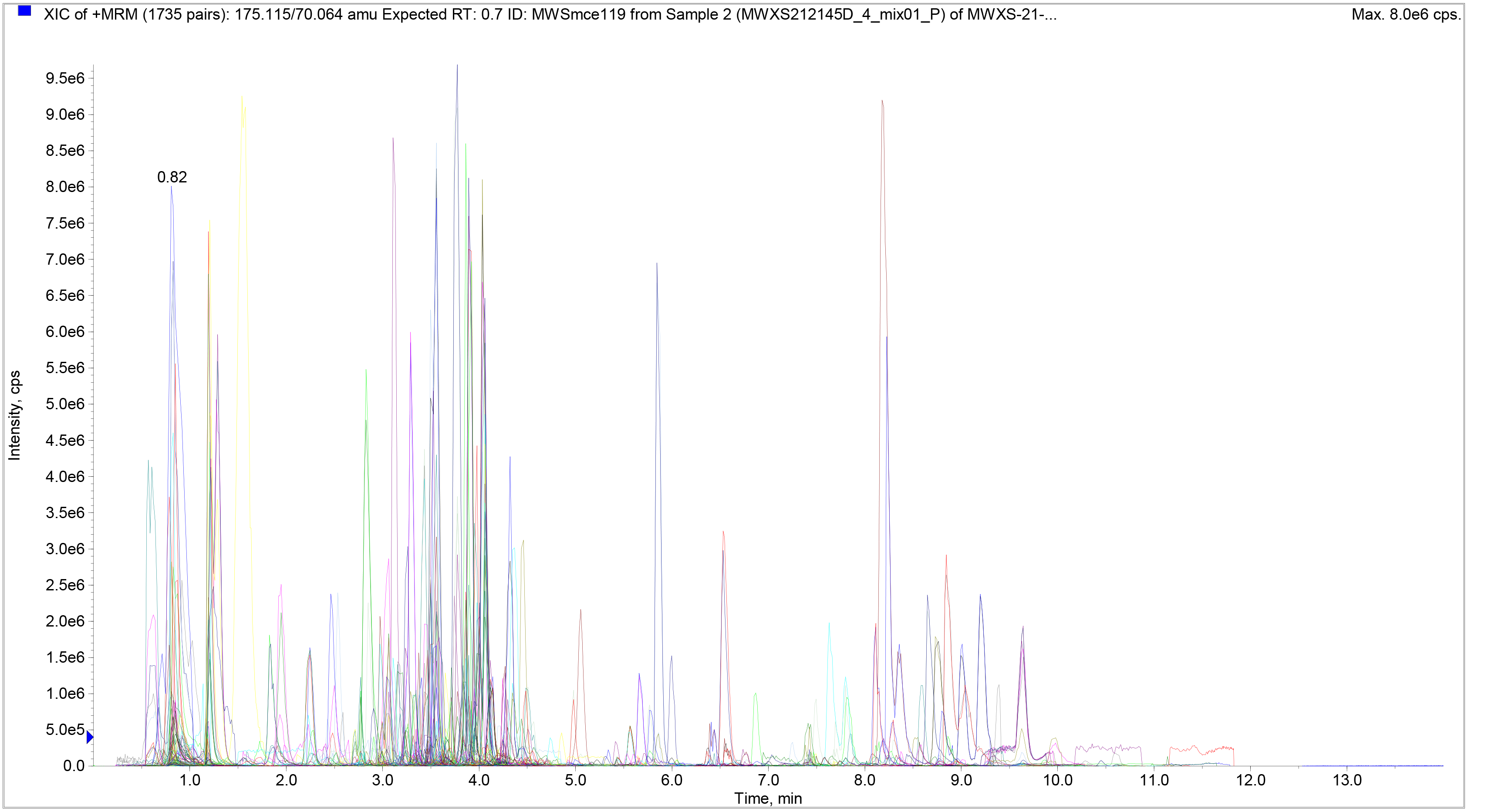

Supplement: Supplementary file 15 [file Image_6.TIF]

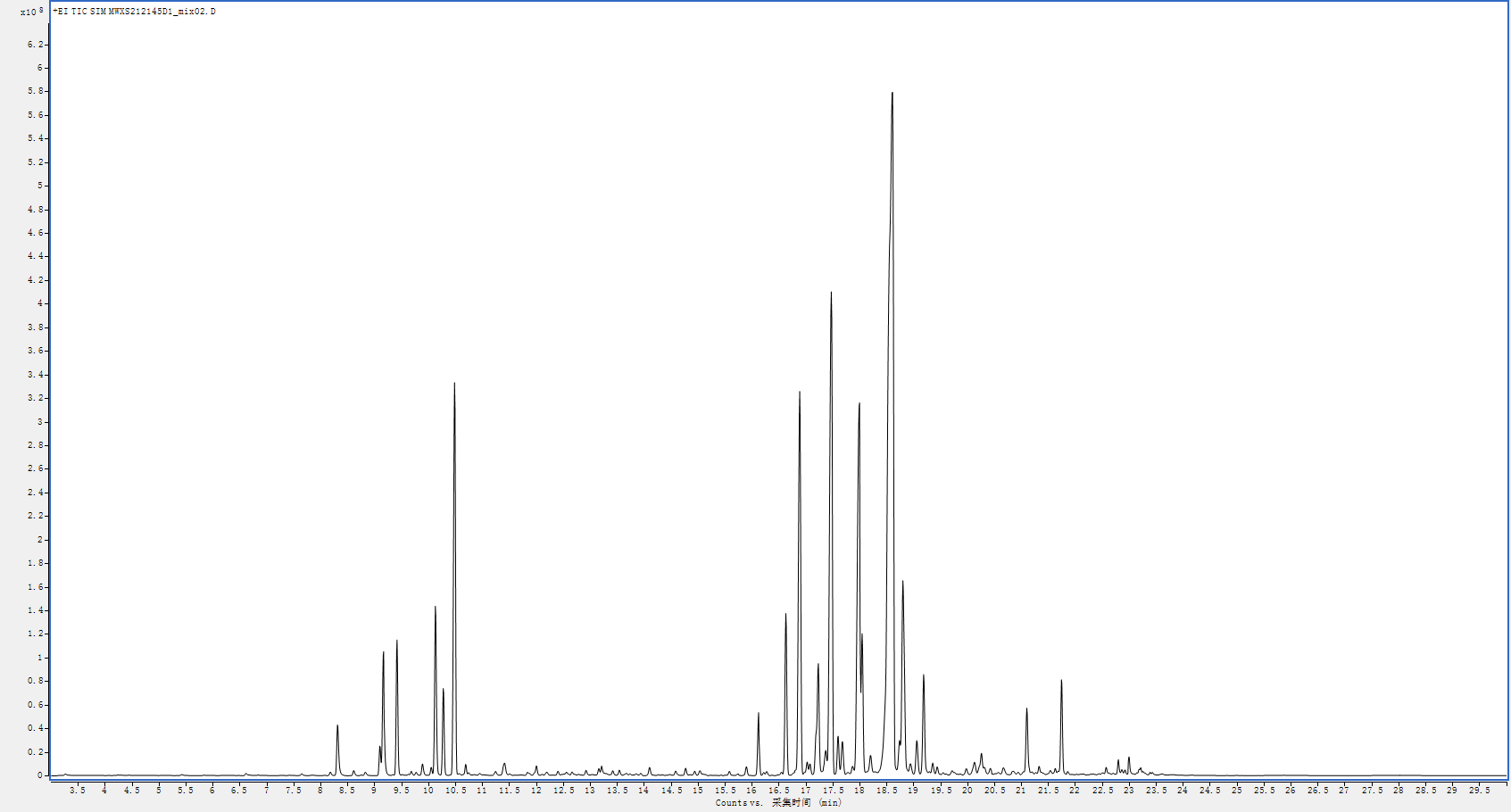

Supplement: Supplementary file 16 [file Image_7.TIF]

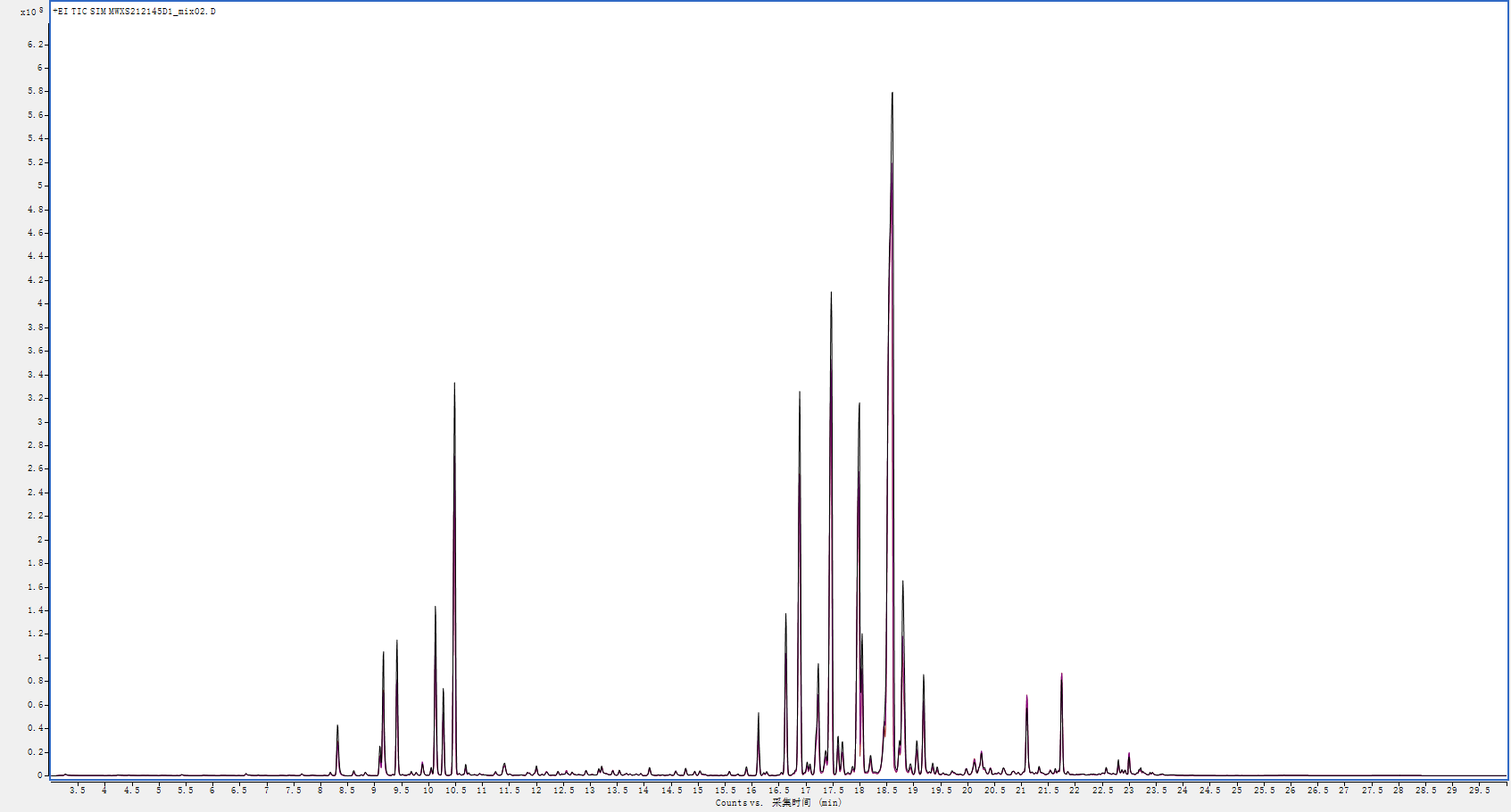

Supplement: Supplementary file 17 [file Image_8.TIF]

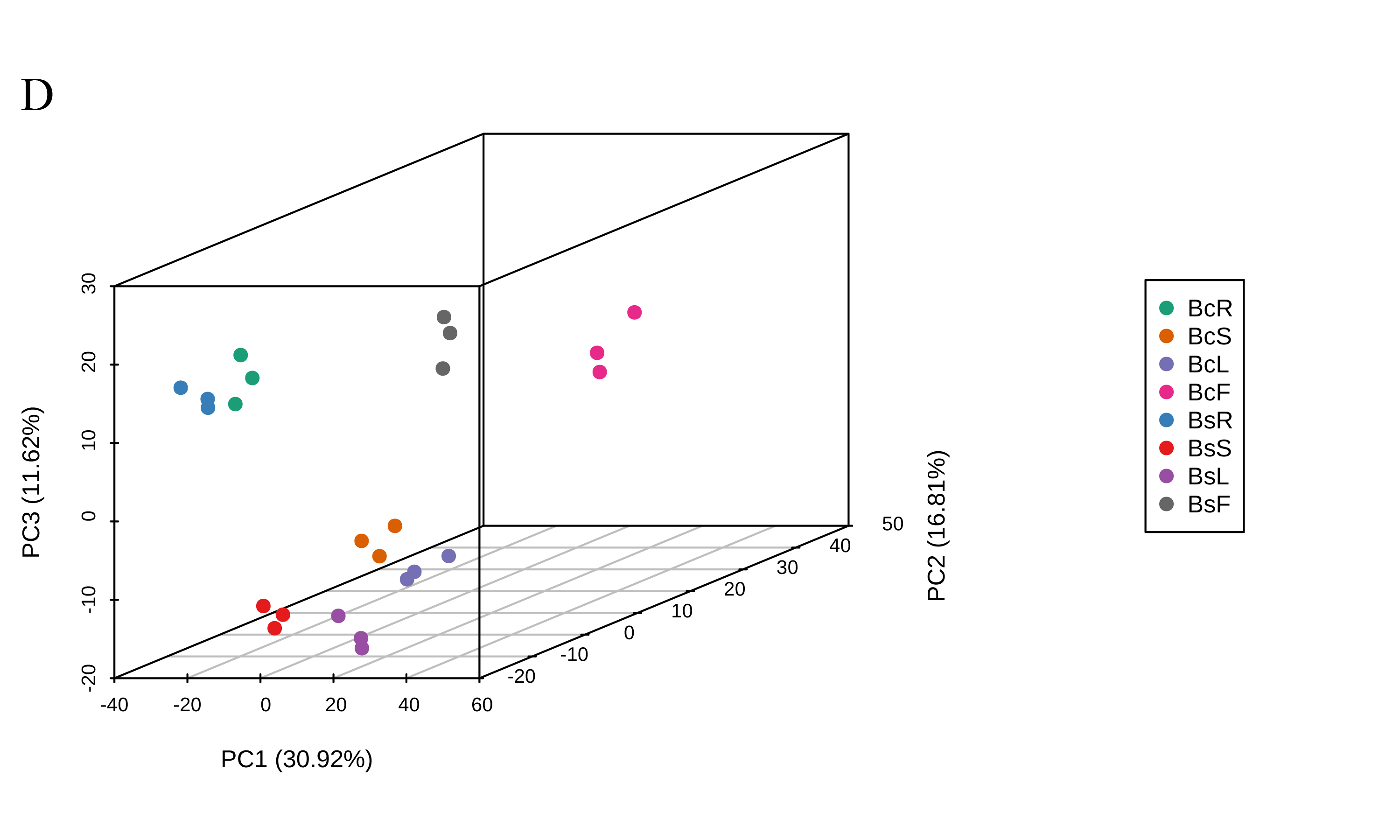

Supplement: Supplementary file 18 [file Image_9.TIF]
